# *Scedosporium* Infection in Recipients of Kidney Transplants from Deceased Near-Drowning Donor

**DOI:** 10.3201/eid2911.231000

**Published:** 2023-11

**Authors:** Devprakash Choudhary, Harsimran Kaur, Vanji Nathan Subramani, Smita Pattanaik, Shivakumar S. Patil, Jasmine Sethi, Manharpreet Kaur, Priya Sreenivasan, Sheetal Thakur, Parul Gupta, Arvind Sekar, Sarbpreet Singh, Muralidharan Jayashree, Deepesh Kenwar, Shivaprakash M. Rudramurthy, Ashish Sharma

**Affiliations:** Postgraduate Institute of Medical Education and Research, Chandigarh, India

**Keywords:** fungi, donor-derived fungal infection, respiratory infections, *Scedosporium aurianticum*, pneumonia, near-drowning organ donor, kidney transplant recipients, voriconazole, India

## Abstract

*Scedosporium aurianticum* infection developed in 2 recipients of kidney transplants in India, acquired from the same deceased near-drowning donor. Given the substantial risk for death associated with *Scedosporium* infection among solid-organ transplant recipients, safety protocols for organ transplantation from nearly drowned donors should be thoroughly revaluated and refined.

Drowning causes 236,000 deaths annually worldwide and is the third leading cause of accidental child death (*1*). Hospitalization from near-drowning occurs 2–20 times more frequently than fatal drownings (*2*). Near-drowning can result in *Scedosporium* spp. fungal infection, which causes pneumonia with a high mortality rate among nearly drowned children and young adults (*2*). Detecting *Scedosporium* in deceased persons is challenging, and infections thus often remain undetected. Because donor-derived *Scedosporium* infections (DDSI) from nearly drowned donors (NDD) have been linked to substantial allograft loss and increased risk for death among kidney transplant recipients (*3*–*6*), undetected *Scedosporium* poses a substantial concern when considering that person for organ donation. 

We report on 2 kidney transplant recipients from an NDD, probably infected with *Scedosporium aurianticum*. The Postgraduate Institute of Medical Education and Research ethics committee approved the study. We obtained informed consent from both case-patients to ensure understanding and voluntary participation. 

A 2-year-old girl weighing 15 kg was admitted to hospital with hypoxic ischemic encephalopathy and respiratory distress after a nonfatal near-drowning experience in a water tank. Her fever persisted despite antimicrobial treatment for suspected pneumonia but resolved after subsequent liposomal amphotericin B therapy. The girl was declared brain dead after 2 weeks of hospitalization, and her kidneys were retrieved for transplantation (Appendix). 

Recipient 1, a 42-year-old woman, received 1 kidney from the deceased NDD. However, 10 days after the procedure, thrombosis developed in the graft renal artery, necessitating a graft nephrectomy. The allograft exhibited septate fungal hyphae, and grew *Scedosporium* on culture (Figure). She received a 6-month course of voriconazole and remained symptom-free on hemodialysis while awaiting a second transplant. (Appendix). 

**Figure:**
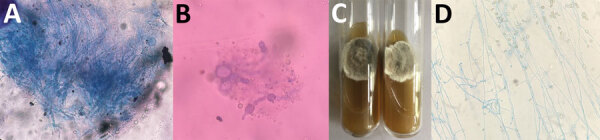
Testing for *Scedosporium aurianticum* infection in 2 recipients of kidney transplants from deceased near-drowning donor, India. A, B) Potassium hydroxide mount of renal allograft tissue from transplant recipient 1 (A) and skin biopsy from transplant recipient 2 (B) showing septate hyphae. C) Culture on Sabouraud dextrose agar showing a greyish-white colony of *S. aurianticum* from recipients 1 (left) and 2 (right). D) Lactophenol cotton blue mount from a culture from recipient 1 showing smooth-walled sessile conidia on cylindrical or flask-shaped conidiogenous cells.

Recipient 2, a 23-year-old woman who received the other kidney from the same NDD, developed high-grade fever 3 days after transplantation. We suspected fungal infection on the basis of high β-D-glucan despite sterile blood cultures and initiated liposomal amphotericin B therapy. However, after we identified *S. aurianticum* infection in the first recipient, we switched the second patient’s treatment to voriconazole. We briefly halted voriconazole therapy because of a period of elevated liver enzymes, during which the patient experienced occasional headaches and swelling developed in her left leg. Microscopy of The aspirate from the swelling revealed *S. aurianticum* mold (Figure), and amplified fragment-length polymorphism molecular typing (Appendix Figure 2) suggested a likely acquisition by common kidney donor. After recipient 2 resumed voriconazole therapy, her swelling resolved, and she remained well with stable graft function 12 months after the kidney transplant (Appendix Figure 1). 

Donor-derived infection occurs in 0.2%–1.7% of solid organ transplant recipients (*7*). However, because of the unique characteristics of drowning, ubiquitous fungi of genus *Scedosporium* can permeate the donor’s respiratory system, increasing risk of transmission to transplant recipients. We searched transplant literature for additional accounts of probable DDSI cases on the basis of the uniform definition of donor-derived infections from a NDD (*7*). DDSI from NDD poses an unusually heightened risk of death among solid organ transplant recipients (*3*–*6*). Consequently, transplant centers remain cautious about considering organs from NDDs (*8*).

*Scedosporium* has emerged as the predominant fungal pathogen causing pneumonia after near-drowning events (*2*). The International Society for Human and Animal Mycology (https://www.isham.org) recently introduced a distinct category for fatal cerebral infections after near-drowning incidents linked to *Scedosporium*, which has been documented to precipitate potentially fatal disseminated infections in 70% of immunocompetent and 100% of immunocompromised hosts (*9*). Addressing *Scedosporium* infection is particularly challenging because of its inherent antifungal resistance, propensity for rapid spread (notably from the lungs to the central nervous system), limited sensitivity of culture based methods, and relatively slow growth of cultured isolates compared to other common saprophytic molds. Those factors collectively lead to delayed diagnoses, elevated therapeutic failures, and increased relapse rates (*10*). 

The risk of infection transmission during drowning events is influenced by several factors, including the type of drowning. Dry drowning, in which the airways close due to spasms without fluid inhalation to the lungs, often results in better outcomes compared with other types. Water temperature also plays a role; cooler temperatures are often linked to more favorable results. Other considerations affecting risk include volume of aspirated water; occurrence of gastric aspiration, which can harm pulmonary epithelial barriers; and specifics of the drowning location, such as water depth, with shallow water presenting a higher risk (*2*,*8*). 

Although the Disease Transmission Advisory Committee of the Organ Procurement and Transplant Network/United Network for Organ Sharing (https://unos.org) has been operational for more than a decade, specific risk factors for DDSI transmission from NDDs have not yet been defined. The passive reporting system used by the Disease Transmission Advisory Committee and frequent omissions of crucial NDD data in donor medical records impede comprehensive understanding of DDSI transmission risks (*7*). Furthermore, identifying DDSI from NDDs before transmission poses substantial challenges, such as selecting effective and accurate detection methods and determining the samples needed for testing and optimal time for collection. 

Routine PCR screening of organs from NDDs for fungi would ensure accurate identification, timely detection, prompt management, and well-informed decision-making. In addition, uniform international guidelines regarding use of organs from NDDs are needed to address critical technical and procedural issues essential for mitigating risk for DDSI transmission. 

Appendix Additional information about study of *Scedosporium* infection in kidney transplant recipients from deceased near-drowning donor
