# Supplementary material for: Scedosporium Infection in Recipients of Kidney Transplants from Deceased Near-Drowning Donor
Source: Emerg Infect Dis. 2023 Nov;29(11):2406–8. doi: 10.3201/eid2911.231000 (PMC10617326; doi:10.3201/eid2911.231000)
Supplement: Appendix — Additional information about study of Scedosporium infection in kidney transplant recipients from deceased near-drowning donor [file 23-1000-Techapp-s1.pdf]

Article DOI: https://doi.org/10.3201/eid2911.231000
EID cannot ensure accessibility for supplementary materials supplied by authors. Readers who have difficulty accessing supplementary content should contact the authors for assistance.

Scedosporium Infection in Recipients of Kidney Transplants from Deceased Near-Drowning Donor

Appendix

Deceased Donor Clinical Parameters

Donor presented with compensated shock with normal CT head. Meropenem and vancomycin were initiated to treat possible polymicrobial infections. Persistent fever(39.5-40°C) on day-five of admission which resolved after starting LAMB. Over two-weeks, her bronchoalveolar lavage, blood, and urine cultures remained sterile with fluctuating leucocyte (3,100-11,160/mm3), and norepinephrine (0.1mcg/kg/min) was required to achieve mean arterial pressure>50th-centile. Day-11 CT-head showed cerebral edema, and brain death was declared on day-14. Only kidneys were retrieved.

Kidney Transplant Recipient 1

On maintainance hemodialysis for five year for endstage renal disease due to chronic glomerulonephritis underwent KT receiveing standard antimicrobial prophylaxis and immunosuppressive regimen. Within a week post-KT, KTR-1 creatinine decreased from 9 to 5.2 mg/dl. On day-10, anuria and absent graft blood flow led to graft-nephrectomy for renal-artery thrombosis. Post graft nephrectomy she was discharged with a normal MRI brain and sterile blood cultures. She continued hemodialysis and completed a six-month oral voriconazole(200mg BD) course, maintaining therapeutic levels 5-5.2µg/ml. Follow-up- Nine months

Kidney Transplant Recipient 2

On maintainance hemodialysis for six year for endstage renal disease due to chronic glomerulonephritis underwent KT receiveing standard antimicrobial prophylaxis and immunosuppressive regimen. Three days post-KT, she developed persistent fever(38.5-39°C),

leukopenia(2,500µL), and thrombocytopenia(70,000µL). Her treatment was switched from LAMB to oral voriconazole(200 mgBD) when *Scedosporium* was identified in KTR-1. Voriconzole course was temporarily interrupted due to elevated liver enzymes, her blood cultures were sterile and brain imaging was normal. Two-months later, voriconazole was resumed at reduced dosage(100mg BD). At nine-month follow-up therapeutic voriconazole levels(4.2-4.5µg/ml) are maintained with a serum creatinine (SCr) level of 1.34mg/dl.

Immunosuppression Protocol Kidney Transplant Recipients 1 and 2

Standard antimicrobial-prophylaxis (cefuroxime-1.5gm), intraoperative methylprednisolone (500mg), anti-thymocyte globulin (ATG-150mg X 3-days), and a standard triple-drug regimen of immunosuppressive (tacrolimus [5mgBD], mycophenolate-mofetil [1gmBD], and prednisolone [25mgOD])

Mycology

The allograft of KTR-1 and leg-aspirate of KTR-2 exhibited septate fungal hyphae under calcofluor KOH wet-mount examination (Figure 1). Both samples cultured on Sabouraud dextrose-agar yielded greyish-brown molds at 25°C and 37°C, identified as *Scedosporium* spp based on smooth-walled sessile conidia on cylindrical or flask-shaped conidiogenous-cells (Figure 1). Further sequencing of the rDNA's internal transcribed spacer region confirmed the isolates as *Scedosporium aurianticum*. Both isolates showed a 100% match with the *S. aurianticum* FMR 8630 type culture and were submitted to GenBank (accession numbers OQ891109 and OQ891110). Amplified fragment length polymorphism (AFLP) typing indicated a 99% match between isolates (Appendix Figure 2), confirming common-donor origin. Both isolates have been stored in the National Culture Collection of Pathogenic Fungi, Chandigarh, India. Antifungal susceptibility testing per clinical and laboratory standards institute (CLSI, 2017) broth microdilution-technique (M38) displayed varying minimum inhibitory concentrations (MIC-µg/ml): amphotericin B (4–8), voriconazole (0.06–0.12), itraconazole (4 each), Posaconazole (2 each), caspofungin (16 each), anidulafungin (4–8), and micafungin (8–16).

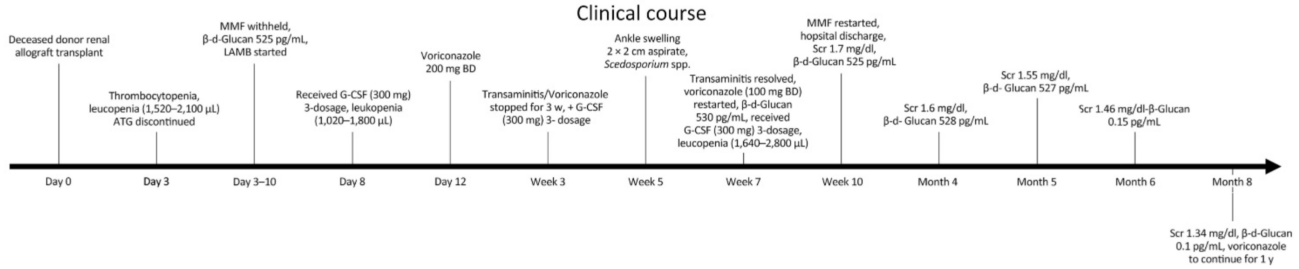

Appendix Figure 1. Clinical course of kidney transplant recipient 2.

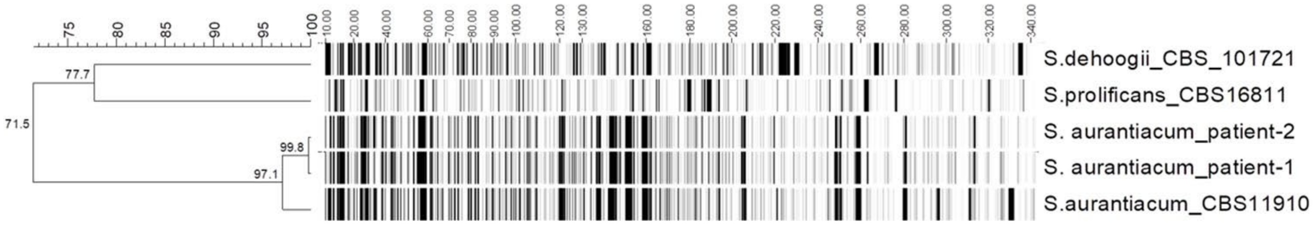

Appendix Figure 2. Amplified fragment length polymorphism of isolates from both patients, *S. aurianticum* CBS (Centraalbureau voor Schimmelcultures) 11910, *Lomentospora prolificans* CBS 16811, and *S. dehoogii* CBS 101721.
